# Exploring CCR5 + T regulatory cell subset dysfunction in type 1 diabetes patients: implications for immune regulation

**DOI:** 10.1007/s12026-024-09508-2

**Published:** 2024-06-27

**Authors:** Ławrynowicz Urszula, Juhas Ulana, Słomiński Bartosz, Okońska Maja, Myśliwiec Małgorzata, Ryba-Stanisławowska Monika

**Affiliations:** 1 Department of Medical Immunology, Faculty of Medicine, Medical University of Gdańsk, Gdańsk, Poland; 2 Division of Bioenergetics and Physiology of Exercise, Faculty of Health Sciences, Medical University of Gdańsk, Gdańsk, Poland; 3 Department of Paediatrics, Diabetology and Endocrinology, Faculty of Medicine, Medical University of Gdańsk, Gdańsk, Poland

**Keywords:** T regulatory cell, Type 1 diabetes, Immune regulation

## Abstract

T regulatory lymphocytes (Treg) expressing CCR5 exhibit strong suppression activity in various autoimmune disorders. However, there remains a lack of comprehensive understanding regarding their involvement in the development of type 1 diabetes (T1D). In this study, we examined the role of the CCR5/CCL5 axis in regulating inflammatory response and its impact on regulatory T cells in type 1 diabetes (T1D). We hypothesize that dysregulation of the CCR5/CCL5 axis contributes to the development and progression of T1D through modulation of Treg-dependent immune responses. We analyzed the expression levels of CCR5 on Tregs isolated from individuals with T1D, as well as the plasma concentration of its main ligands. We found that Tregs from T1D patients exhibited decreased expression of CCR5 compared to healthy controls. Additionally, we observed a correlation between the expression levels of CCR5 on Tregs and their immunosuppressive function in T1D patients. Our results indicate the impaired migratory capacity of CCR5 + Tregs, suggesting a possible link between the dysregulation of the CCR5/CCL5 axis and impaired immune regulation in T1D. In line with previous studies, our findings support the notion that dysregulation of the CCR5/CCL5 axis contributes to the development and progression of type 1 diabetes (T1D) by modulating Treg-dependent immune responses. The decreased expression of CCR5 on Tregs in T1D patients suggests a potential impairment in the migratory capacity of these cells, which could compromise their ability to suppress autoreactive T cells and maintain immune homeostasis. Furthermore, our study highlights the importance of CCR5 as a biomarker for identifying dysfunctional Tregs in T1D.

## Introduction

Type 1 diabetes (T1D) is an autoimmune disease characterized by the selective destruction of pancreatic β-cells. It is caused by the inflammatory reaction in and around Langerhans islets. Along with the disease progression, systemic inflammation and increased leukocyte migration into tissues occur, leading to impaired blood flow, local inflammation, and the development of micro- and macrovascular injuries [1, 2]. In recent years, there has been an increased understanding of the immunological, genetic, and environmental basics of the development and progression of T1D and its complications [3]. However, the immunological triggers and the exact pathogenesis of T1DM still remain unclear [4]. It is well demonstrated that chemokines and their receptors play a significant role in orchestrating cellular infiltrations into inflamed tissues in T1D [5, 6]. Chemokines are released mainly by the local endothelium and by invading immune cells. These chemoattractant inflammatory mediators regulate a broad spectrum of important biological processes involved in the pathogenesis of T1D such as inflammatory response, chemotaxis, homeostasis, and angiogenesis [7]. There is an extensive network of chemokines that attract migrating leukocytes expressing the corresponding chemokine receptors. These include the chemokine receptor type 5 (CCR5) involved in intracellular signaling and its main ligand C–C motif chemokine ligand 5 (CCL5), also called RANTES (Regulated on Activation, Normal T-cell Expressed and Secreted). CCR5 is best known as a major coreceptor for primary macrophage-tropic HIV-1 strains and its natural ligands are RANTES (CCL5), monocyte chemoattractant protein-1 (MCP-1 also called CCL2), macrophage inflammatory protein 1α (MIP-1α/CCL3), and macrophage inflammatory protein 1β (MIP-1β/CCL4). CCR5 is expressed on activated Th1 cells, monocytes, macrophages, dendritic and NK cells, and microglial and endothelial cells [8]. CCR5 participates in the regulation of proinflammatory response by modulating the behavior, survival, and retention of immune cells in tissues [9]. The low-grade inflammation that underlies the course of T1D plays a critical role in the development of micro- and macrovascular diabetic complications [10, 11].

Previous studies have demonstrated that chemokines can directly regulate the migration and recruitment of cells to injury sites of inflammation. All CC-chemokines contain nuclear factor-kappa B (NF-kB) binding motifs, and their expression is significantly upregulated under inflammatory conditions [12, 13]. CCL2, CCL3, CCL4, and CCL5 may be induced by an inflammatory stimulus. The increased expression of CCL5 mediates the arrest and transmigration of monocytes/macrophages into the damaged endothelium by binding with its receptor CCR5, which is involved in the inflammatory response to endothelial injury [12, 14, 15]. Furthermore, CCR5/CCL5 appears to affect the suppressive function of T-regulatory lymphocytes (Tregs), which play an important role in maintaining immune homeostasis [16]. Activation of Treg cells leads to up-regulation of CCR5 receptors on their surface. It has been shown that Treg cells with CCR5 expression represent strong suppressive activity in many autoimmune diseases [17]. However, their role in the pathogenesis of T1D is not fully understood yet.

The function of the CCR5 receptor in the cellular immune response suggests that alteration in its sequence or expression may contribute to the initiation and progression of diabetic microvascular complications [17–19]. CCR5 encoding gene is highly polymorphic, with one of the most intensely studied alterations being the 32-base pair deletion (CCR5-Δ32) [20]. In individuals homozygous for CCR5-Δ32, the receptor is not present on a cell surface. At the same time in heterozygous individuals, the CCR5 surface expression is reduced by over 50% when compared to the wild-type counterparts [21].

Our previous work revealed that the presence of Δ32 allele in T1D patients is associated with increased inflammation and a significantly more severe course of the disease. The findings of our studies suggest that carriers of CCR5-Δ32 polymorphism are at higher risk of developing microvascular complications such as diabetic retinopathy [22] as well as concomitant autoimmune disorders — celiac disease and inflammatory thyroid disease [23]. However, the mechanism of this correlation remains unclear. Therefore, we hypothesized that the interaction of CCL5/RANTES with CCR5 expressed on the immune cell surface may be pivotal in the process of low-grade inflammation and early vascular injury development in T1D juvenile patients. As before, our group revealed the functional defects in the Treg subset in young T1D patients [24] as well as the role of Treg/Th17 imbalance in the progression of microvascular angiopathy [25]. In this study, we focused on the Treg subset, which required further in-depth study.

In the current manuscript, we investigated the involvement of the CCR5/CCL5 axis in the regulation of inflammatory response in T1D and its effect on the quantitative as well as qualitative properties of regulatory T cells. We showed that CCR5 on Tregs is associated with their increased suppressive capacity in vitro. Our results also suggest that the function of CCR5/CCL5 axis in T1D Tregs may be impaired.

## Materials and methods

### Materials

The study group consisted of 120 randomly selected juvenile patients with long-standing (over 1 year) type 1 diabetes mellitus (LS-T1D) recruited from the Clinic of Pediatrics, Department of Diabetology and Endocrinology, Medical University of Gdansk, Poland. Subjects with other autoimmune disorders, microvascular complications, acute inflammatory and infectious diseases, history of hypertension, or blood transfusions were not enrolled in the study. Type 1 diabetes mellitus (T1D) was defined in accordance with American Diabetes Association criteria.

Eight milliliters of venous peripheral blood from each individual was collected into Vacutainer tubes containing heparin (BD Bioscience, USA). The blood glucose level, biochemical measurement of renal function, lipid status, and glycosylated hemoglobin (HbA1c) were measured at the time of sampling. In all examined patients, the C-peptide levels were below 0.5 ng/ml. All patients were treated with humanized insulin. The control group consisted of 45 age and sex-matched healthy individuals with no signs of autoimmune, chronic, inflammatory, or neoplastic disease at the time of sampling and no evidence of T1D in their families.

The study followed the principles of the Declaration of Helsinki and was approved by The Ethics Committee of The Medical University of Gdansk (NKBBN/645/2019). From all parents of juvenile participants, written informed consent was obtained. A summary of the clinical characteristics of the analyzed subject groups is presented in Table 3.

### Reagents and antibodies

The following monoclonal antibodies (mAbs) were used in cytometric studies (fluorochromes and clones in brackets): anti-CD3 (PE-Cy7, SK7), anti-CD4 (APC or PE-Cy5, RPAT4), anti-CD25 (PE, 2A3), anti-CD127 (PE-Cy7, AO19D5). Items were purchased from BD Biosciences, USA, or BioLegend, USA. Intracellular staining for Foxp3 (FITC, 206D) and IFN-γ (PerCP-Cy.5.5, B27) was performed with ready-to-use kits according to the manufacturer’s suggestions (BioLegend, USA and BD Biosciences, USA, respectively). Additionally, the following reagents were used for the stimulation of cell cultures: anti-CD3 (clone UCHT1, eBioscience, USA), anti-CD28 (clone CD28.2, eBioscience, USA) antibodies, rIL-2 (BioLegend, USA), PMA (Sigma, Poland), ionomycin (Sigma, Poland), and GolgiStop (BD Biosciences, USA).

### PBMC isolation and cell culture

Eight milliliters of venous peripheral blood from each individual was collected in Vacutainer tubes containing heparin (BD Bioscience, USA). Peripheral blood mononuclear cells (PBMCs) were isolated from the donors’ blood by density gradient centrifugation over Histopaque (Sigma-Aldrich, Poland) and cultured on 24-well plates in RPMI 1640 (Sigma-Aldrich, Poland) supplemented with 5% heat-inactivated fetal bovine serum (FBS; Sigma-Aldrich, Poland) and 1% penicillin/streptomycin (Sigma-Aldrich, Poland).

For each study individual, the isolated PBMCs were placed in two wells. First, the control well (starting point) was designated “non-stimulated” and second “stimulated.” Cells in both wells were treated with anti-CD3 and anti-CD28 antibodies, both at a concentration of 5 µg/ml. Wells designated as “stimulated” were additionally treated with recombinant human CCL5 at a 15 ng/ml concentration. Cells in both wells were also stimulated with recombinant human IL-2 (100 U/ml). Cultures were incubated at 37 °C with 5% CO2 for 48 h and then cells were designated for cytometric analysis.

### Isolation and culture of CD4 + CD25 + CD127low Tregs

CD4 + CD25 + CD127low Tregs were separated from PBMC by a two-step magnetic separation procedure using EasySep™ Human CD4 + CD127lowCD25 + Regulatory T Cell Isolation Kit (Stem Cell, USA). First, we used EasySep Releasable RapidSpheres along with the CD25 Positive Selection Cocktail to isolate CD25 + cells. Next, we utilized a CD4 + T-cell enrichment cocktail to ensure the exclusive isolation of CD4 + CD25 + cells. The resulting cell suspension underwent further refinement by adding CD127high Depletion Cocktail and Dextran RapidSpheres, allowing for the depletion of CD127 + cells. The final isolated fraction consisted of highly purified CD4 + CD25 + CD127low cells. The purity of isolated cells was assessed by flow cytometry. Starting with fresh peripheral blood, the CD4 + cell content of the enriched fraction was typically 94 ± 5% and the purity of selected CD4 + CD127lowCD25 + Tregs ranged from 86 to 96%.

Magnetically selected CD4 + CD25 + CD127low T cells were then cultured on the round bottom 96-well plates. For each study individual, the isolated Tregs were placed in two wells. First, the control well (starting point) was designated “non-stimulated” and second “stimulated.” Cells in both wells were treated with anti-CD3 and anti-CD28 antibodies, both at a concentration of 5 µg/ml. Wells designated as “stimulated” were additionally treated with recombinant human CCL5 at a 15 ng/ml concentration. Cells in both wells were also stimulated with recombinant human IL-2 (100 U/ml). Cultures were incubated at 37 °C with 5% CO2 for 48 h. At the same time, from each study individual, the autologous “non-stimulated” PBMCs were isolated by density gradient preparation over Histopaque-1083 (Sigma) and cultured for 48 h in RPMI medium supplemented with 5% heat-inactivated FBS.

### Qualitative characterization of regulatory T cells

In order to determine the immunosuppressive activity of Tregs, the IFNγ suppression assay was performed. The quality of stimulated Tregs was checked after 48 h of culture. The assay started by making co-cultures of Tregs with autologous PBMCs in a 1:1 ratio. For each co-culture, 50,000 isolated Tregs, as well as PBMCs, were used. The stimulation consisted of 50 ng/ml of phorbol 12- myristate 13-acetate, 500 ng/ml of ionomycin, and 2 ml/ml of GolgiStop (BDBiosciences, USA). After 5 h, the cells were stained to CD3 + CD4 + IFN-c + phenotype and analyzed by flow cytometry.

### Chemotaxis assay

Treg-cell trafficking was assessed by using a trans-well assay to measure the specific chemotactic activity. A total of 10^6^ freshly isolated PBMCs were suspended in 200 µL RPMI and transferred into the upper chambers of 6.5-mm diameter, 5.0-µm pore-size polycarbonate membrane filter trans-well plates (Costar Corning, Cambridge, MA). RPMI (600 µL) or RPMI + CCL5 (15 ng/ml, Biologend) were added to the lower chamber. After 3 h at 37 °C, migrated cells were collected in the lower chamber, stained with anti-CD4 and anti-CD25, anti-CD127, anti-FOXP3, and anti-CD195 mAbs, and analyzed by flow cytometry analysis. The chemotactic index for Treg cells (CI-Treg) was defined as the ratio of percentages of Treg cells in CD4 + T cells of migrated cells and percentages of Treg cells in CD4 + T cells of original PBMCs.

### Flow cytometric analysis

Cells were harvested and labeled by monoclonal antibodies against the following surface and intracellular markers: CD3, CD4, CD25, CD127, CD195 (CCR5), FOXP3, and IFN-γ. All samples were incubated in the dark for 20 min at room temperature, washed with 2 mL PBS, and centrifuged twice (1500 rpm, 6 min). Cells stained for intracellular markers were incubated with ready-to-use fixation buffer (BioLegend, USA) for 20 min in the dark at room temperature. Next, washed twice with 1 ml of freshly prepared permeabilization buffer (BioLegend, USA). Intracellularly stained cells were suspended in 100 µl of permeabilization buffer and labeled with antibodies in the same conditions as previously described. After incubation, labeled cells were washed twice with 1 ml of permeabilization buffer. All stained samples were resuspended in 400 µL PBS.

**Fig. 1:**
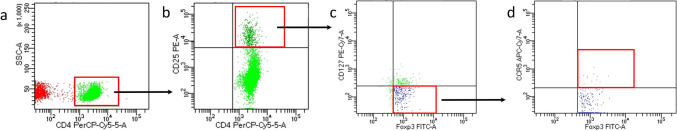
Gating strategy used to analyze Treg and CCR5 + Treg cells in PBMC. Identification of the total viable cell count of lymphocytes was assessed firstly based on forward (FSC) and side (SSC) light scatter parameters profiles designating size and granularity, respectively. Within a single, live, lymphocyte population, CD4 + lymphocytes were selected (Fig. 1a). This CD4 + cell population was further gated into CD4 + cells with the highest level of CD25 fluorescence defined as CD4 + CD25high cells (Fig. 1b). Events from the later gate are then transposed to the CD127 vs. FOXP3 dot plot. Cells with CD127-/low FOXP3 + profile were defined as a Treg cell population (Fig. 1c). Lastly, in the Treg population, those with FOXP3 + /CCR5 + profile were defined as a CCR5 + Treg cell population (Fig. 1d)

Expression of cell surface and intracellular markers was assessed using flow cytometry (LSRII, Becton Dickinson, USA) after gating on live cells determined by scatter characteristics. Data were analyzed by FACSDiva 6.0 Software (Becton Dickinson, USA). Figure 1 shows the gating strategy used for the analysis of regulatory T cell subsets.

### Plasma concentration of CCR5 ligands

Plasma was separated from the whole blood samples by centrifugation (2500 rpm/15 min) from all the patients and stored at − 20 °C. The plasma concentration of MCP-1, MIP-1B, and RANTES in the T1D patients (*n* = 48) and healthy controls (*n* = 22) were quantified using a commercial Quantikine HS ELISA test from R&D Systems (USA).

### Statistics

All statistical analyses were performed using Statistica 13.0 (StatSoft, Inc., USA) using either the nonparametric Wilcoxon test or the Mann–Whitney *U* test if not stated otherwise, with *P*-values less than 0.05 defined as significant. Figures were created with GraphPad Prism 8.

## Results

### T1D patients exhibit a significantly lower fraction of CCR5 + Tregs than healthy individuals

We previously demonstrated that discrepancies in *CCR5* genotype may be associated with the worsened course of T1D, greater risk of diabetic retinopathy, and comorbidities such as celiac disease and autoimmune thyroiditis [22, 23]. In order to characterize possible mechanisms responsible for the previously described observations, we compared CD4 + CD25highFoxp3-expressing Tregs isolated from healthy controls and diabetic patients for the cellular expression of the CCR5 receptor.

**Fig. 2:**
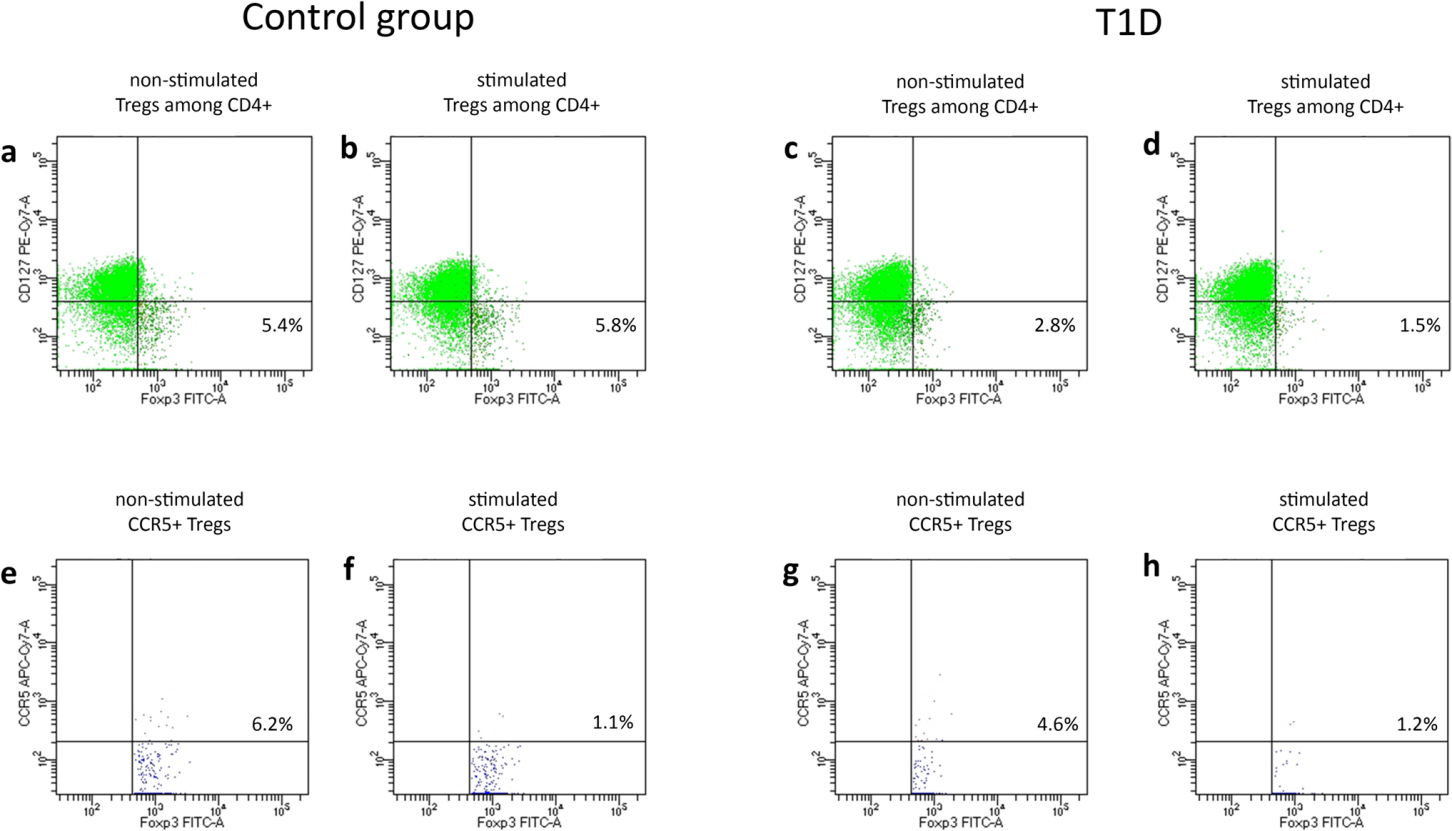
Representative flow cytometric analysis of Treg (**a**–**d**) and CCR5 + Treg (**e**–**h**) cells before and after in vitro CCL5 treatment. The percentage of CD4 + CD25high CD127low Foxp3 + among CD4 + T cells was determined by flow cytometry. 50,000 events were acquired. Gate on Treg and CCR5 + Treg was established as described above. After gating on CD4 + CD25high cells, the frequency of non-stimulated (**a**, **c**) and CCL5 stimulated (**b**, **d**) CD4 + CD25highFoxp3 + cells was determined

**Fig. 3:**
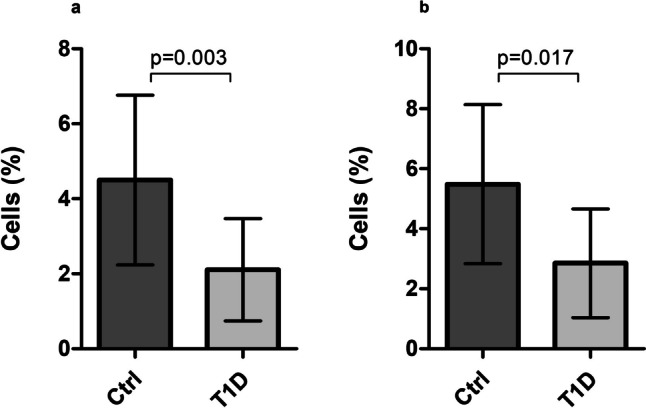
Quantitative characterization of Treg lymphocyte subsets. Fraction of CD4 + CD25highCD127-FOXP3 + Tregs among CD4 + lymphocytes (**a**) and CCR5 + Tregs among CD4 + CD25highCD127-FOXP3 + Tregs (**b**). Data are presented as medians and standard deviations (SD). Box height corresponds to the median and whiskers to SD

**Fig. 4:**
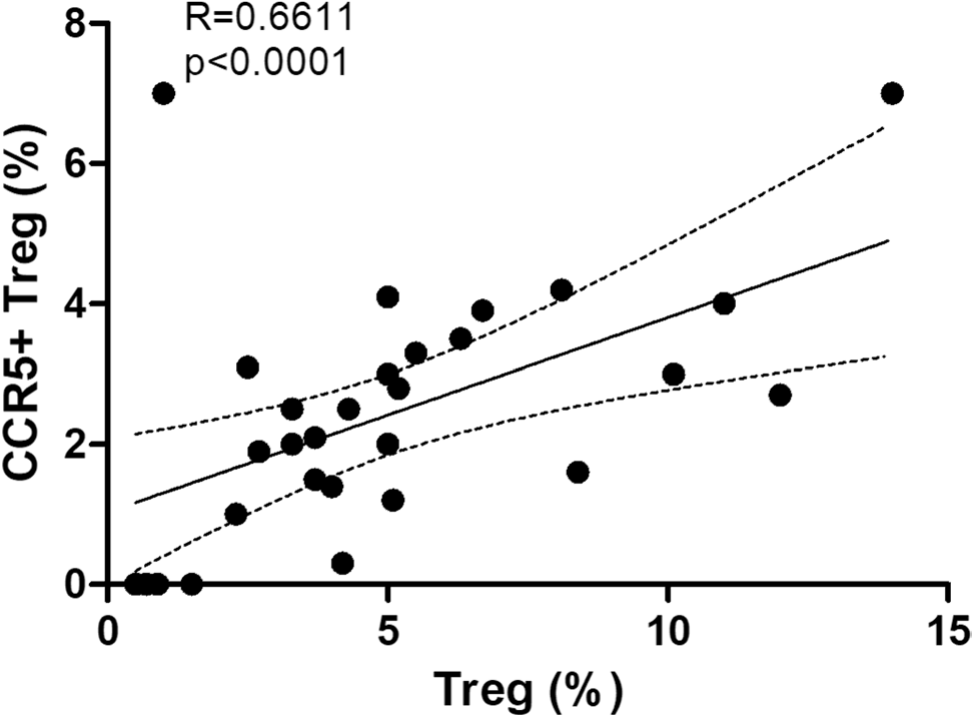
Association between the number of CCR5 + Treg and Treg cells in T1D. The Spearman test was used to calculate the strength of correlation; *R* = 0.6; *p* < 0.01

**Fig. 5:**
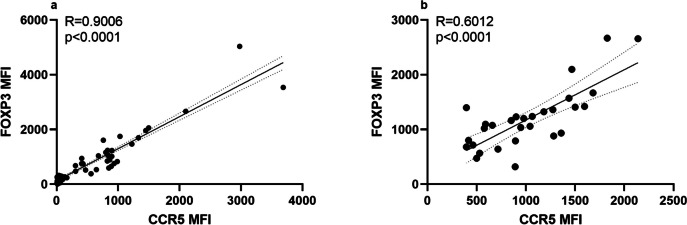
Correlation of FOXP3 and CCR5 expression in Treg cells of analyzed T1D subjects. During the cytometric analysis of regulatory T cells, the relative expression of CCR5 and FOXP3 on/in these cells respectively was quantified as a ratio of mean fluorescence intensity (MFI) for FOXP3 or CCR5 to MFI for appropriate isotype control. The Spearman test was used to calculate the strength of correlation. **a** Correlation of FOXP3 and CCR5 expression in/on resting cells. Spearman correlation; *R* = 0.9; *p* < 0.01. **b** Correlation of FOXP3 and CCR5 expression in/on CCL5-stimulated cells. Spearman correlation; *R* = 0.6; *p* < 0.01

PBMC were isolated from venous blood, and after 48 h of culture, they were subjected to cytometric analysis to assess the number and fraction of regulatory T cells and Treg cells with the expression of CCR5 receptor (Fig. 2). The results indicated that Treg and Treg expressing CCR5 were significantly depleted in the type 1 diabetes group (Fig. 3). Table 1 summarizes fractions of Treg subsets in the T1D and control groups. Furthermore, in T1D patients, lack or decreased expression of CCR5 was associated with significantly lower numbers of T regulatory cells (Fig. 4). Interestingly, analysis of CCR5 expression presented as mean fluorescence intensity showed a strong correlation between the expression of the CCR5 receptor and the transcription factor FOXP3 (Fig. 5).

**Table 1:** Quantitative characterization of Treg lymphocyte subsets

Group	The percentage of CD4 + CD25highCD127-FOXP3 + (%)*	*p*	FOXP3 MFI	*p*	CCR5 MFI	*p*
T1D	2.25 ± 1.32	**0.003**	539	**0.008**	-	-
(*n* = 42)						
Control	4.78 ± 2.87		645		-	
(*n* = 21)						
Group	The percentage of CD4 + CD25highCD127-FOXP3 + CCR5 + (%)**	*p*	FOXP3 MFI	*p*	CCR5 MFI	*p*
T1D	3.650 ± 1.84	**0.017**	1284	0.180	712	0.062
(*n* = 42)						
**Control**	6.1 ± 2.62		1314		684	
**(*n* = 21)**						

Data are presented as medians and standard deviations.

All the differences were calculated by the Mann–Whitney *U* test.

*MFI* mean fluorescence intensity.

*The percentage of cells among CD4 + lymphocytes.

**The percentage of cells among CD4 + CD25highCD127-FOXP3 + cells.

### CCL5 treatment affects the suppressive capacity of Tregs in T1D in vitro

**Fig. 6:**
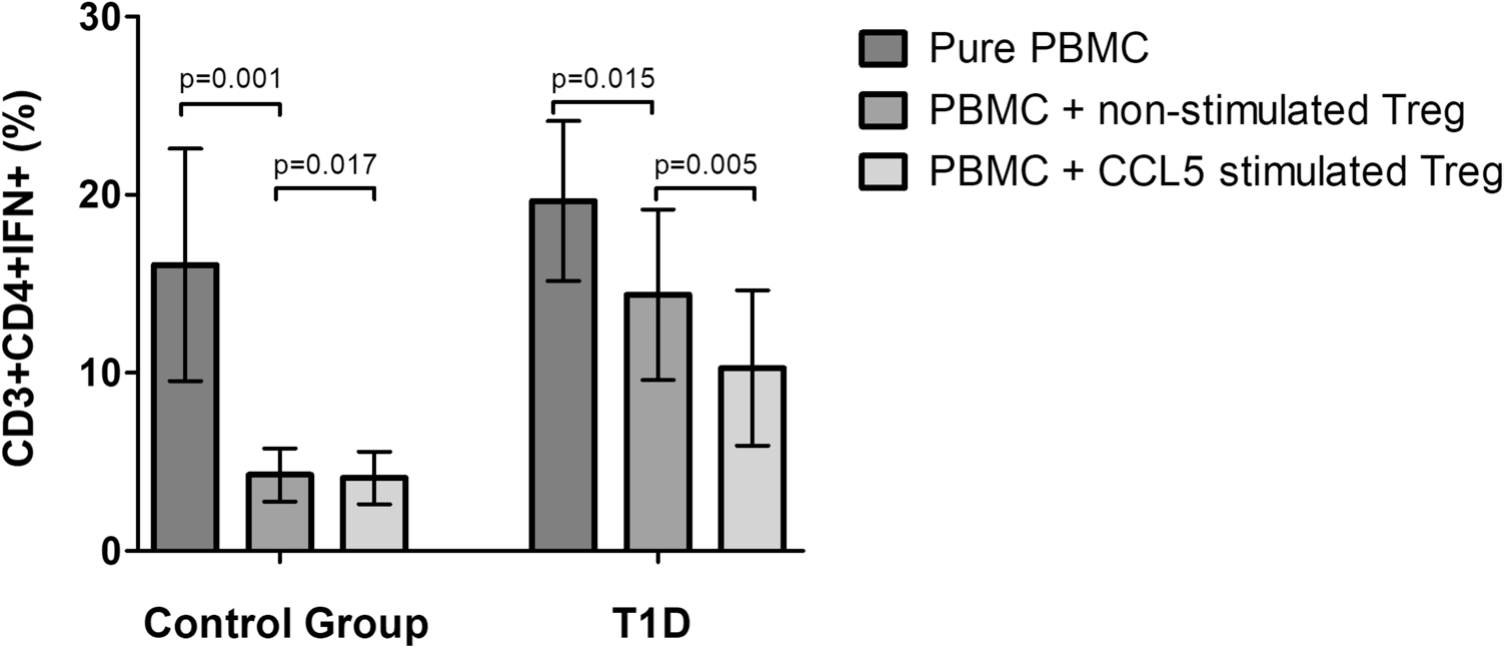
The frequency of CD3 + CD4 + IFN-γ + (%). Treg cells were isolated from analyzed groups of individuals — T1D and the control group. Then resting or CCL5-stimulated Tregs were cultured with autologous PBMC and IFN-gamma suppression assay was performed in order to determine the percentage of IFN-γ secreting T cells. Data were calculated with the nonparametric Wilcoxon test and are presented as medians and SD

To determine whether the observed positive correlation between CCR5 and FOXP3 expression levels on Treg cells treated in vitro with CCL5 affects the qualitative characteristics of these cells, we examined the effect of CCL5 treatment on the immunosuppressive function of Treg subpopulation. Magnetically selected CD4 + CD127lowCD25 + regulatory T cells after 48 h in vitro culture with or without CCL5 were added to isolated autologous PBMCs in a 1:1 ratio for another 24 h. The IFN-γ suppression assay was performed in order to determine the ability of Tregs to suppress the production of proinflammatory IFN-γ by the effector T lymphocytes (Teff). The fraction of effector T lymphocytes producing IFN-γ (CD3 + CD4 + IFN-γ +) was significantly lower when Tregs were treated with CCL5 in comparison to non-CCL5-stimulated Tregs. The analysis revealed that in patients with type 1 diabetes (*n* = 42) CCL5 treated Tregs more efficiently suppressed the production of IFN-γ by effector T lymphocytes compared to Tregs “not-stimulated” with CCL5 (Fig. 6). The fraction of CD3 + CD4 + IFN-γ + effector T lymphocytes among PBMCs was lower when Tregs were treated with CCL5 in comparison to resting (non-CCL5-stimulated) Tregs. In the healthy, control group (*n* = 21), the frequency of effector T lymphocytes lowered only when Tregs were added to the culture of PBMCs and practically did not change upon CCL5 stimulation (Fig. 6).

### Reduced migratory response of CCR5 + Tregs to CCL5 in T1D

We noted that CCL5 can significantly affect the properties of Tregs. Therefore, we investigated whether the CCR5/CCL5 axis functions properly in type 1 diabetes patients in terms of Treg lymphocyte migration. For this purpose, trans-well plates with a membrane pore size of 5 µm were used. The chemotactic index for Tregs was determined as the ratio of the percentage of Tregs among cells migrating under the influence of CCL5 compared to unstimulated control (spontaneous migration).

The average chemotactic index for CCR5 + Treg cells migrating toward CCL5 was 2.78 ± 0.87 for the Control group (*n* = 7), whereas for T1D patients (*n* = 3) it was 1.95 ± 0.72. Due to the low number of patients in this experiment, the results are limited and the difference was not statistically significant (*p* = 0.052); however, it may suggest that Treg CCR5/CCL5-dependent capacity to migrate to the inflammation site may be impaired in T1D (Table 2).

**Table 2:** Migration assay

Group	Chemotactic index	*p*-value
T1D (*n* = 3)	1.95 ± 0.72	**0.052**
Control (*n* = 7)	2.78 ± 0.87	

### Plasma levels of CCR5 ligands change with disease progression

To extend our previous results showing that patients with longstanding T1D presented significantly elevated levels of CCR5 ligand – CCL2 [22], we decided to determine if plasma concentrations of CCR5 ligands are related to the duration of the disease. We evaluated the concentration of three main CCR5 ligands in patients with newly diagnosed type 1 diabetes (T1D-ND), and long-standing type 1 diabetes (T1D-LS), as well as the healthy control group.

**Fig. 7:**
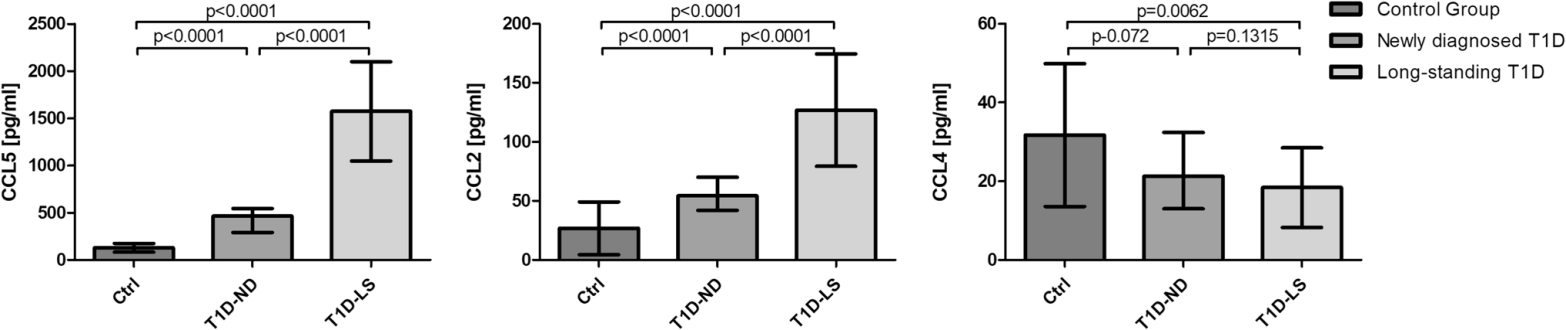
Plasma levels of selected CCR5 ligands in the control group, T1D-ND and T1D-LS patients. Plasma was isolated and frozen at the time of blood sample processing. Subsequent determination of concentrations of selected chemokines was performed using ELISA assays. Data are presented as medians and SD. The Mann–Whitney *U* test was used to calculate the differences between the groups

CCL5 and CCL2 chemokines were shown to be significantly elevated in the type 1 diabetes group vs. controls (Fig. 7). Moreover, high concentrations of these chemokines were correlated with the duration of diabetes. In contrast, the concentration of CCL4, which is attributed to a protective effect against the development of type 1 diabetes [26], was reduced in T1D patients compared to the control group, although without statistical significance. All results are presented in Fig. 7.

## Discussion

The chemokine network plays an essential role in immune responses ranging from immune cell infiltration to its activation. It is well established that CC chemokine receptor 5 (CCR5) is expressed mainly by CD4 + T helper 1 (Th1) cells and significantly affects the regulation of type 1 (Th1) immune response [27]. However, it is also expressed by other cell types including macrophages, dendritic cells, and endothelial and epithelial cells. Its main ligand CCL5/RANTES, although initially considered to be T cell-specific chemokine, may interact with a broad spectrum of cells and it is an important component of multiple biological processes [28, 29]. Evidence on the role of CCR5/CCL5 axis in the regulation of inflammatory response is conflicting. It is generally accepted that CCR5 and its ligands mediate proinflammatory actions through the recruitment of activated leukocytes. On the other hand, CCR5/CCL5 axis may promote endothelial repair [30]. According to Dobaczewski et al., CCR5 surface expression appears to identify mononuclear cell subsets with anti-inflammatory properties. Several studies have shown that CCR5 is constitutively expressed on Tregs in humans and likely provides Tregs with a competitive advantage over naïve T cells to migrate more efficiently to the periphery [31, 32]. However, there is no data demonstrating the role of this chemokine receptor and its ligands in Treg function in human type 1 diabetes. In the present study, we sought to assess the connection between the CCR5/CCL5 axis and quantitative as well as qualitative properties of Treg cells in T1D.

Our results showed that T1D patients exhibited a significantly lower fraction of CCR5 + Tregs than healthy individuals. Moreover, the absence of CCR5 was associated with significantly lower numbers of T-regulatory cells in diabetic patients. Intracellular flow cytometry analysis revealed that the CCR5 + subset of Tregs expresses higher levels of FOXP3. In addition, FOXP3 expression increased along with the expression of CCR5, which suggests that the CCR5/CCL5 axis may not only influence quantitative but also qualitative characteristics of Treg in T1D.

It is important to note that the results obtained for other autoimmune models are contradictory. Soler et al. obtained similar results in psoriasis and showed that psoriatic CCR5 + Tregs cells are both numerically and functionally deficient compared to healthy controls [32]. On the other hand, Gellatly et al. revealed that Tregs expressed significantly higher levels of CCR5 during vitiligo progression. What’s more, CCR5 + Tregs in human skin also expressed type 1 proinflammatory genes like TBX21 and IFNG, thus it is possible that Tregs may lose their suppressive status and use CCR5 to promote the progression of the disease. In addition, the authors found that CCL5/CCR5 axis functioned as a chemokine circuit between effector T cells and Tregs in vitiligo patients [33].

Taking that all into consideration, we have tried to define the in vitro effect of CCL5 on the suppressive potential of magnetically sorted Tregs from patients with type 1 diabetes. To check this, we tested the ability of Tregs to inhibit the production of IFN-γ by effector CD3 + CD4 + T lymphocytes in mixed co-cultures. The frequency of CD3 + CD4 + IFN-γ + cells was significantly lower in co-cultures with CCL5 pre-stimulated Tregs when compared to non-stimulated ones. However, the suppressive activity of T1D Tregs under CCL5 treatment was not sufficient to fully inhibit the production of IFN-γ by effector T cells. Although our initial results suggested that CCR5 expressed on regulatory T cells may be associated with better suppressive effects in vitro, our subsequent results showed that CCR5 + Tregs in T1D diabetes are functionally deficient in suppression. This is in line with previous reports that confirmed that regulatory T cells in patients with T1D show decreased suppressive capacity [24, 34, 35].

Emerging evidence suggests that the CCR5/CCL5 axis may play an important role in Tregs trafficking to inflammation sites in cancer, infections, and autoimmune diseases. In melanoma, CCR5/CCL5 recruits Tregs with high TGFβ1 expression [36]. In gastric and esophageal cancer, CCL5 secreted by lymph node-derived cells recruits CCR5 + Tregs to the tumor lesion [37]. CCR5 + Treg cells have been experimentally shown in the periphery to limit Th1 immune responses in murine inflammatory disease models including Leishmania major infection [38] and acute graft-versus-host disease [39, 40]. Zhang et al. demonstrated that CCR5 is essential for Treg’s initial migration to the inflammatory allograft environment [41]. Therefore, we assessed the migration efficiency of both control and diabetic CCR5 + Treg toward CCL5. The average chemotactic index for CCR5 + Treg cells migrating toward CCL5 was higher for the control group than for the T1D group. Our results suggest that the CCR5/CCL5-dependent Treg recruitment to inflammation site in patients with type 1 diabetes may be impaired; however, a larger group of patients needs to be assessed to confirm this observation.

In our previous study, we demonstrated a significantly elevated plasma concentration of MCP-1 in T1D patients [22]. Thus, we decided to compare plasma levels of different CCR5 ligands in the group of patients with newly diagnosed T1D, long-standing T1D (over 1 year), and healthy controls. CCL5 and CCL2 chemokines were shown to be significantly elevated in both T1D groups and substantially higher in T1D-LS. In contrast, the concentration of the chemokine CCL4 was reduced in patients compared to the healthy subjects, however, the differences were not statistically significant. This is in line with the study of Meagher et al., which postulated the protective role of CCL4 in the development of type 1 diabetes [26]. However, recently, Chang et al. demonstrated that CCL4 is upregulated to modulate the downstream inflammatory cytokines in type 1 DM [42].

Decreased expression of CCR5 on the surface of Treg cells and lower migratory response to CCL5 stimulation may be an effect of desensitization to this chemokine due to its high plasma concentration in T1D patients (Table 3). Neel et al. described mechanisms behind chemokine receptor desensitization and internalization. Constant exposure of chemokine receptors to ligands may result in their degradation and prolonged desensitization. According to the literature, CCR5 can undergo up to 80% internalization when subjected to its ligands. This effect persists during continuous exposure to the ligand. However, after the removal of RANTES, internalized CCR5 recycles to the cell surface and its function as well as sensitivity to ligands is restored [43, 44].

**Table 3:** Clinical characteristics of T1D patients and plasma levels of CCR5 ligands

	T1D	Control group	*p*-value
Sex (F/M)	54/66	27/18	0.115
Age (years)	14.25 ± 3.86	15.65 ± 3.24	0.123
Duration of the disease (years)	3.66 ± 2.82	–	–
BMI	21.79 ± 4.97	19.4 ± 2.06	0.166
HbA1c (%)	8.38 ± 1.45	–	–
CCL5 (pg/ml)	1433.09 ± 554.72	553.31 ± 130.51	**0.0004**
CCL2 (pg/ml)	95.96 ± 59,74	55.98 ± 27.31	**0.0086**
CCL4 (pg/ml)	10.36 ± 20.25	18.61 ± 20.81	0.067

These findings suggest a possible explanation for the impaired suppressive capacity of Treg cells in T1D. Reduced surface expression of CCR5 may be responsible for the reduced homing of diabetic Treg toward inflammatory signals (including CCL5). In addition, the lower expression level of FOXP3 in CCR5 + Tregs in T1D may contribute to their inability to sufficiently suppress the expansion of pathogenic effector T cells, which may contribute to shifting the balance toward inflammatory Th17 cells as it was previously described by us [25]. Our results allow for a better understanding of the biology of Tregs which will have implications in other inflammatory and autoimmune diseases.

## Data Availability

The data that support the findings of this study are available on a reasonable request from the corresponding author [U.Ł.]. The data are not publicly available due to containing information that could compromise the privacy of research participants.
